# Over-Expressed Twist Associates with Markers of Epithelial Mesenchymal Transition and Predicts Poor Prognosis in Breast Cancers via ERK and Akt Activation

**DOI:** 10.1371/journal.pone.0135851

**Published:** 2015-08-21

**Authors:** Yong-Qu Zhang, Xiao-Long Wei, Yuan-Ke Liang, Wei-Ling Chen, Fan Zhang, Jing-Wen Bai, Si-Qi Qiu, Cai-Wen Du, Wen-He Huang, Guo-Jun Zhang

**Affiliations:** 1 The Breast Center, Cancer Hospital of Shantou University Medical College, Shantou, Guangdong, China; 2 Cancer Research Center of Shantou University Medical College, Shantou, Guangdong, China; 3 Department of Pathology, Cancer Hospital of Shantou University Medical College, Shantou, Guangdong, China; 4 Changjiang Scholar’s Research Laboratory, Cancer Hospital of Shantou University Medical College, Shantou, Guangdong, China; 5 Department of Breast Medical Oncology, Cancer Hospital of Shantou University Medical College, Shantou, Guangdong, China; Wayne State University School of Medicine, UNITED STATES

## Abstract

Overexpression of Twist, a highly conserved basic helix-loop-helix transcription factor, is associated with epithelial-mesenchymal transition (EMT) and predicts poor prognosis in various kinds of cancers, including breast cancer. In order to further clarify Twist’s role in breast cancer, we detected Twist expression in breast cancer tissues by immunohistochemistry. Twist expression was observed in 54% (220/408) of breast cancer patients and was positively associated with tumor size, Ki67, VEGF-C and HER2 expression. Conversely, Twist was negatively associated with estrogen receptor (ER), progesterone receptor (PgR) and E-cadherin expression. Patients with Twist expression had a poorer prognosis for 30-month disease free survival (DFS) (82.9%) than patients with negative Twist (92.3%). Overexpression of Twist led to dramatic changes in cellular morphology, proliferation, migratory/invasive capability, and expression of EMT-related biomarkers in breast cancer cells. Moreover, we show that Twist serves as a driver of tumorigenesis, as well as an inducer of EMT, at least in part, through activation of the Akt and extracellular signal-regulated protein kinase (ERK) pathways which are critical for Twist-mediated EMT. Our results demonstrate that Twist expression is an important prognostic factor in breast cancer patients.

## Introduction

Breast cancer is the most frequently diagnosed cancer and accounts for 22.9% of all cancers in women worldwide[1]. In 2008, approximately 1.38 million (23%) new breast cancer cases were diagnosed in women and 458,503 deaths occurred from breast cancer, comprising 13.7% of all female cancer deaths[1]. Factors that increase the risk of a recurrence include young age[2], lymph node metastasis[3], high histology grade[4], microcalcifications on mammography[5], high nuclear grade, high mitotic count, high Ki-67 proliferative index[6], absence of hormone receptors (estrogen or progesterone receptors) [7], certain molecular subtypes(a five-biomarker panel:estrogen receptor, progesterone receptor, HER-2, CK5/6, and epidermal growth factor receptor)[8] and the lack of adjuvant therapy[9]. Several oncoproteins or tumor suppressors including HER-2 and p53 have also been found to predict clinical outcome or prognosis of breast cancer, and Twist has already been used as a prognostic marker in cervical cancer[10], bladder and prostate cancers[11] and chronic kidney disease[12]. However, the mechanism underlying how Twist predicts prognosis is not well defined in breast cancers.

Epithelial-mesenchymal transition (EMT) is a process by which epithelial cells lose their orientation and cell-cell contacts, and acquire the migratory and invasive properties of mesenchymal cells. In addition to its crucial role in the differentiation of many tissues and organs[13], EMT has also been shown to cause organ fibrosis and promote carcinoma progression through a variety of mechanisms. Twist is a highly conserved basic helix-loop-helix transcription factor and is one of the most important factors regulating EMT[14, 15]. Twist acts as an oncogene and is overexpressed in a variety of cancers, including breast[16], lung[17], prostate cancers[18, 19], and gastric carcinoma[20]. Twist upregulates N-cadherin and downregulates E-cadherin expression, and induces EMT[21]. Furthermore, Twist plays an important role in some physiological processes, including metastasis[21], angiogenesis and chromosomal instability[22].Twist is negatively associated with p53 protein and protects cancer cells from apoptotic cell death[23].

In addition, Twist performs a vital role in generation of drug resistance against chemotherapeutic drugs, such as Taxol[24], cisplatin and doxorubicin[25]. Recently, targeting Twist by RNAi or chemotherapeutic drugs has been shown to successfully inhibit cancer growth[26]. Moreover, several inhibitors of antagonizing Twist signaling have also been identified[27, 28]. A variety of signal transduction pathways can activate Twist expression, including Wnt signaling[29, 30], Akt[31], signal transducer and activator of transcription 3 (STAT3)[32], and mitogen-activated protein kinases (MAPKs), which increase Twist protein levels in breast cancer cells[33, 34]. In breast cancer patients, overexpression of Twist correlates with cancer development and progression through decreasing E-cadherin expression. In a recent study, overexpression of Twist was associated with poorer prognosis in both HER-2 overexpressed and triple negative breast cancers. Thus, this study aims to further evaluate Twist’s role in breast cancer development and progression and to identify the potential molecular mechanisms.

## Materials and Methods

### Cell lines and reagents

Human breast cancer cell lines MCF-7, T47D, SKBR3, MDA-MB-231, and BT549 human breast carcinoma cells were obtained from the American Type Culture Collection (Manassas, USA). All cells were cultured at 37°C and 5% CO_2_ in Dulbecco’s modified Eagle’s medium (DMEM) supplemented with 10% fetal bovine serum.

The mouse monoclonal antibodies used were Twist, p27, β-actin. Rabbit polyclonal antibodies used were E-cadherin, vimentin, Snail, Akt, phospho-Akt (Ser473), ERK1/2, p-ERK1/2 (Thr-202/Tyr-204), GSK3β and p-GSK3β (Ser-9). Antibodies used in this study are summarized in Table 1.

**Table 1:** Antibodies used in the study.

Antigen	Source	Clone No.	Manufacturer
Twist	Mouse	10E4E6	Abcam
Akt	Rabbit	C73H10	Cell Signaling
p-Akt(Ser473)	Rabbit	193H12	Cell Signal
ERK1/2	Rabbit	polyclonal antibody	Cell Signal
p-ERK1/2(Thr202/Tyr204)	Rabbit	polyclonal antibody	Cell Signal
GSK3β	Rabbit	polyclonal antibody	Bioss
p-GSK3β (Ser-9)	Rabbit	polyclonal antibody	Bioss
Snail	Rabbit	C15D3	Cell Signal
Vimentin	Rabbit	D21H3	Cell Signal
P27	Rabbit	C-19	Santa Cruz
E-cadherin	Rabbit	24E10	Cell Signal
ERα	Rabbit	D8H8	Cell Signal
PR	Rabbit	2F12B4	Labvision
HER2	Rabbit	e2-4001	Labvision
Ki67	Rabbit	8D5	Labvision
VEGF-C	Rabbit	polyclonal antibody	Labvision
P53	Mouse	DO-7	Labvision
β-actin	Mouse	C-4	Santa Cruz

Plasmids pFlag-CMV and pFlag-Twist (a 694-bp human Twist cDNA was inserted into the *Eco*RV/*Xba*I sites of the pFlag-CMV vector) were generously provided by professor Muh-Hwa Yang (National Yang-Ming University). The vector pcDNA3.1-luc2 was obtained from Invitrogen (Carlsbad, CA, USA). Akt inhibitor, LY294002 and ERK inhibitor, U0126 were purchased from Selleck Chemicals (Houston, USA)

### Plasmid DNA transfection

24 hrs prior to transfection, approximately 1 × 10^6^ cells in 10%DMEM without antibiotics were inoculated into a 60 mm dish so that cells will get to 90–95% confluent at the time of transfection. MCF-7 and T47D breast cancer cells were transfected using Lipofectamine 2000 (Invitrogen) with either 5 μg of pFlag-CMV vector as a control or 5 μg of pFlag-Twist according to the manufacturer’s protocol. For stable transfection, the concentration of G418 (Sangon Biotech, Shanghai, China) was determined by drawing a killing curve, and 1 mg/ml of G418 was used to kill non-transfected cells.

### Western blot analysis

Total protein was extracted by lysing cells in RIPA buffer (Cell Signaling Technology, Boston, MA), and protein concentration was determined by Bradford assay. Whole cell extracts were boiled in SDS sample buffer [2% SDS, 50 mmol/L Tris-HCl (pH 6.8), 10% glycerol, 0.002% bromophenol blue, and 6% 2-mercaptoethanol], and denatured proteins were separated by SDS-PAGE. The proteins were transferred onto a polyvinylidene difluoride membrane (Millipore, Billerica, USA), and the membranes were blocked in TBS-T buffer [10 mmol/L Tris-HCl (pH 7.4), 150 mmol/L NaCl, and 0.05% (v/v) Tween 20] containing 5% nonfat dry milk, and probed with primary antibody in blocking buffer at 4°C overnight. Blots were washed 3 times at 10 minutes each with TBS-T buffer and then incubated with secondary antibodies diluted in TBS-T at room temperature for 30 minutes to one hour followed by 3 washes at 10 minutes each. Finally, protein bands were visualized by using Western Chemiluminescent HRP Substrate (Millipore, Billerica, USA) and exposing to X-ray film.

### RNA extraction and quantitative real-time PCR analysis

Total RNA was extracted using TRIzol (Invitrogen, Carlsbad, USA) according to the manufacturer’s protocol. First-strand cDNA was synthesized using a PrimeScript RT Reagent Kit (Takara Bio, Dalian, China) according to the manufacturer’s instructions. Gene expression levels were measured by real-time PCR using SYBR Select Master Mix (Applied Biosystems, New York, USA), and analyzed on a CFX Connect Real-Time system (BIO-RAD, Hercules, USA) according to manufacturer’s instructions. Target gene expression levels in each sample were subsequently normalized to the level of β-actin mRNA. All samples were measured in triplicate. Primers are as follows:

Twist: Forward primer 5ˊ- GTCCGCAGTCTTACGAGGAG-3ˊ; Reverse primer 5ˊ-GCTTGAGGGTCTGAATCTTGCT -3ˊ.

ERα: Forward primer 5ˊ- CTCTCCCACATCAGGCACA-3ˊ; Reverse primer 5ˊ- CTTTGGTCCGTCTCCTCCA -3ˊ.

E-cadherin: Forward primer 5ˊ- AAAGGCCCATTTCCTAAAAACCT-3ˊ; Reverse primer 5ˊ- TGCGTTCTCTATCCAGAGGCT-3ˊ.

Vimentin: Forward primer 5ˊ- GACGCCATCAACACCGAGTT-3 Reverse primer 5ˊ- CTTTGTCGTTGGTTAGCTGGT-3ˊ.

β-actin: Forward primer 5ˊ- TGAGCGCGGCTACAGCTT -3ˊ; Reverse primer 5ˊ- TCCTTAATGTCACGCACGATTT -3ˊ.

### Cell proliferation assay

MCF-7-Twist cells were plated in each well of a 96-well microplate at a density of 2 × 10^3^ cells after transfecting with the plasmid pFlag-Twist for 48 hours. Cells transfected with the pFlag-CMV vector was used as the negative control. Cellular proliferation was determined by a Cell Counting Kit-8 (Beyotime, Jiangsu, China) at Day 1 to Day 6 after transfection. Absorbance was recorded at 450 nm with an ELX800 microplate reader (Bio-Tek, Vermont, USA). Experiments were performed 3 times in triplicate.

### Cell migration and invasion assay

Cell migration assay was performed using BD Falcon Cell Culture Inserts (24-well plate, 8 μm; BD Biocoat, BD Biosciences, Franklin Lake, USA) as described previously[35, 36]. Briefly, the 2 × 10^4^ cells were inoculated in the upper compartment of the cell culture inserts at 42 hours after transfection. The lower chamber was filled with DMEM supplemented with 20% FBS. After 48 hours of incubation at 37°C, non-migrated cells in the upper chamber were removed from the upper surface of the filters using a phosphate-buffered saline (PBS)-soaked cotton swab, and remaining cells were fixed in methanol and stained with 0.1% crystal violet. Cells fixed on the lower face of the chambers were counted under a light microscope. Five high-powered fields were counted for each well and mean numbers of invaded cells per field were counted. All experiments were carried out in duplicate, in 3 independent assays.

For the invasion assay, 4 × 10^4^ cells in DMEM-containing 10% FBS were inoculated in the upper compartment of Matrigel-coated inserts (24-well plate, 8 μm; BD Biocoat, BD Biosciences (Franklin Lake, USA) at 42 hours after transfection, and the lower chamber was filled with DMEM with 20% FBS. At 72 hours after incubation at 37°C, non-invaded cells in the upper chamber were removed from the surface of the filters using a phosphate-buffered saline (PBS)-soaked cotton swab, fixed, stained, and counted as described in cell migration assay.

### Colony formation assay

MCF-7 cells were inoculated at 1 × 10^5^ cells/ml in a 12-well plate and allowed to attach for 24 hours. Cells were then co-transfected with 0.1 μg of pcDNA3.1-Luc2 and 1 μg of pFlag-Twist or 1 μg pFlag-CMV (negative control), using Lipofectamine 2000. At 48 hours post-transfection, the transfected cells were inoculated onto 6 well plates at a density of 800 cells/well with G418 selection (1 mg/ml). After 2–3weeks, cells were stained with Gentian Violet and numbers of colonies were counted and analyzed. The protocol, as reported previously[37]. The experiment was repeated 3 times independently.

### Patients and immunohistochemistry

A total of 408 female patients with breast cancer (median age: 50 years-old; range: 43–59 years-old), treated between August 2011 and October 2013 at the Cancer Hospital of Shantou University Medical College, were enrolled in this study. None of these patients received preoperative treatment, such as chemotherapy or radiotherapy. Correspondingly, 408 primary breast tumor samples, of which 355 were confirmed as invasive ductal carcinoma (87.0%), were fixed in buffered formalin and embedded in paraffin for immunohistochemistry (IHC). Information for clinicopathological features including age, menopausal status, tumor size, nodal status, TNM stage, histology and morphology were collected. TNM stage was classified in accordance with the American Joint Committee on Cancer (AJCC) pathologic tumor-node-metastasis (TNM) classification (seventh edition, 2009). Disease-free survival (DFS) and overall survival (OS) were calculated starting from the date of surgery, until the date of first recurrence or metastasis, or the date of breast cancer-related death. Until the latest follow-up time (March, 2014), either recurrence or metastasis had occurred in 27 of 408 patients (6.6%), and 4 patients had died (1.0%). Written informed consents were obtained for all patients, in particular, for the use of human tissues, and the study protocol was approved by the medical ethics committee of the Cancer Hospital of Shantou University Medical College.

IHC staining for ER, PR, HER2 and Ki67 was routinely performed. Additional immunohistochemistry was performed for Twist, E-cadherin, p53, VEGF-C, p-Akt and p-ERK as previously described [38, 39]. In addition, p-ERK and p-Akt were also immunohistochemically detected in 102 patients with breast cancers. The percentage of positive stained cells was evaluated in the same section of tissue, to analyze the correlation between Twist and p-Akt or p-ERK expression.

### Statistical analysis

Statistical analysis involved use of SPSS 16.0 (SPSS Inc., Chicago, IL). Differences among variables were assessed by χ2 analysis, Spearman's Rank Correlation Test or 2-tailed Student’s t tests. Two-sided P <0.05 was considered statistically significant. Each experiment was done at least in triplicate.

## Results

### Twist regulates the expression of epithelial and mesenchymal markers in human breast cancer cell lines

**Fig 1:**
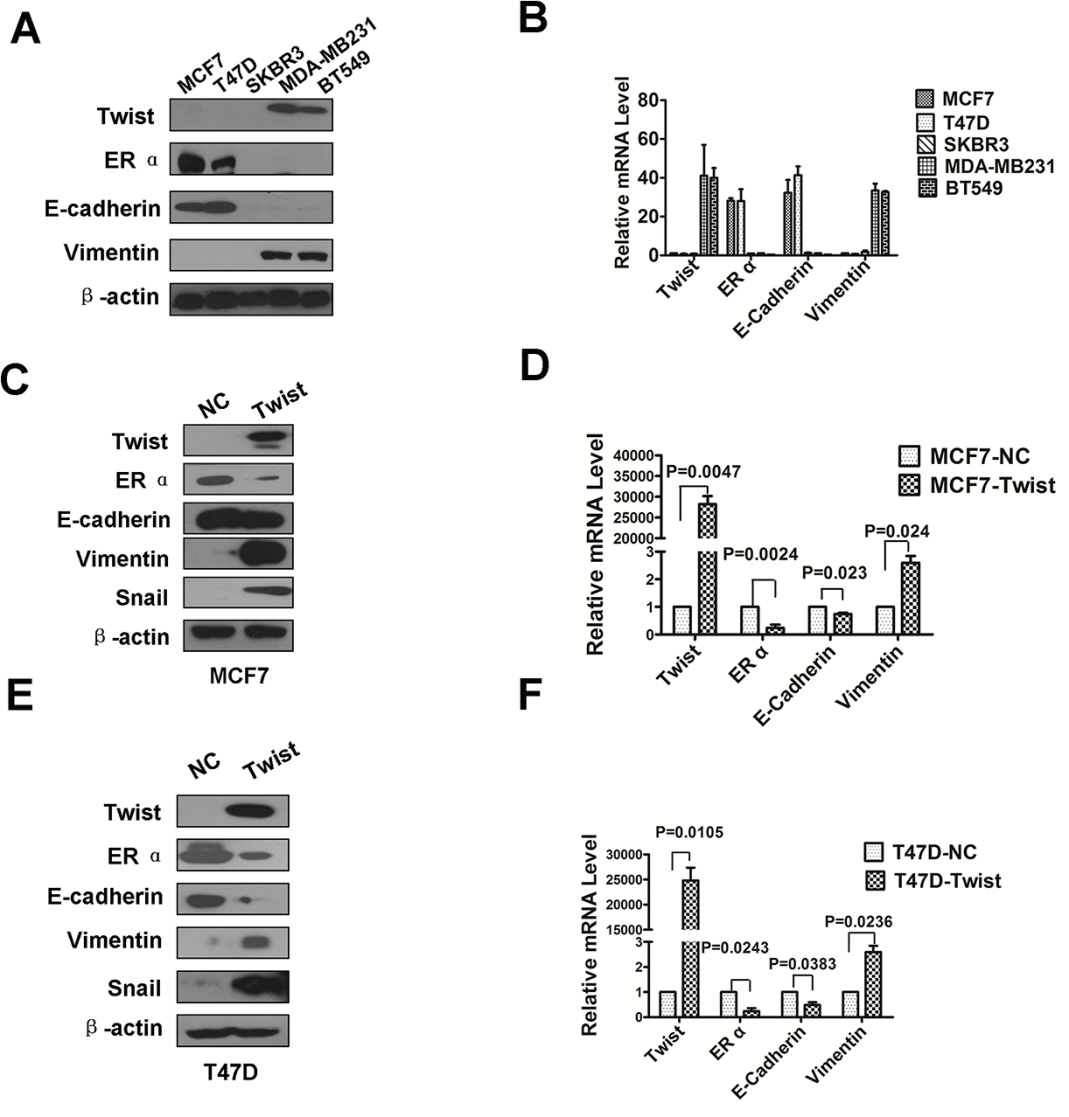
Twist promotes epithelial-mesenchymal transition (EMT) markers. (A) Expression of Twist, ERα, E-cadherin and Vimentin in breast cancer cell lines was analyzed by Western blotting. β-actin was used as a loading control. (B) The transcriptional level of Twist, ERα, E-cadherin and Vimentin were detected by Real-Time PCR analysis. All the data were collected from three independent experiments. (C and E) Overexpression of Twist resulted in a loss of E-cadherin and a gain of Vimentin and Snail in MCF-7 and T47D cells. The expression of ERα, E-cadherin, Vimentin and Snail was detected by western blot analysis. β-actin was used as the loading control. (D and F) Overexpression of Twist resulted in a decrease of E-cadherin and an increase of Vimentin and Snail in mRNA level in MCF-7 and T47D cells. The mRNA expression levels for each target gene were subsequently normalized by the mRNA level of β-actin. All samples were measured in triplicate and *P* value low than 0.05 for MCF-7 and T47D cells overexpressing Twist compared with the corresponding MCF-7 and T47D cells transfected with empty vector. Data shown are means ± SD of at least three independent experiments. *P*-values were obtained using the Student’s *t*-test analysis. Western blot was used to detected Twist and EMT marker expression (n = 3).

To examine the Twist and related EMT marker expression levels, five human breast cancer cell lines were used for Western blot and real-time RT-PCR analysis, of which, MCF-7 and T47D are classified as luminal A, SKBR3 as HER2-overexpressed, and MDA-MB231 and BT549 as triple-negative. Twist protein was highly expressed in the triple-negative breast cancer MDA-MB231 and BT549 cell lines as well as the mesenchymal marker vimentin (Fig 1A). As expected, ERα and epithelial marker E-cadherin were highly expressed in the MCF-7 and T47D Luminal A type breast cancer cell lines. In contrast, ERα and E-cadherin were undetectable in the triple-negative breast cancer cell lines and the HER2-overexpressing SKBR3 breast cancer cell line (Fig 1A). Similarly, quantitative real-time PCR results showed that Twist, ERα, E-cadherin and vimentin mRNA levels were consistent with the protein levels detected by Western blot analysis (Fig 1B).

To explore the role of Twist in breast cancer cells, Twist was ectopically overexpressed in MCF-7 and T47D cells (Fig 1C and 1E) and thereafter the expression of epithelial and mesenchymal markers were detected. We found that overexpression of Twist led to the down-regulation of ERα and E-cadherin, and up-regulation of vimentin and Snail at both the mRNA (Fig 1D and 1F) and protein levels (Fig 1C and 1E). These results demonstrate that overexpression of Twist in MCF-7 and T47D cells caused downregulation of epithelial markers and upregulation of mesenchymal molecules.

### Twist promotes cell proliferation, migration, invasion and colony formation

**Fig 2:**
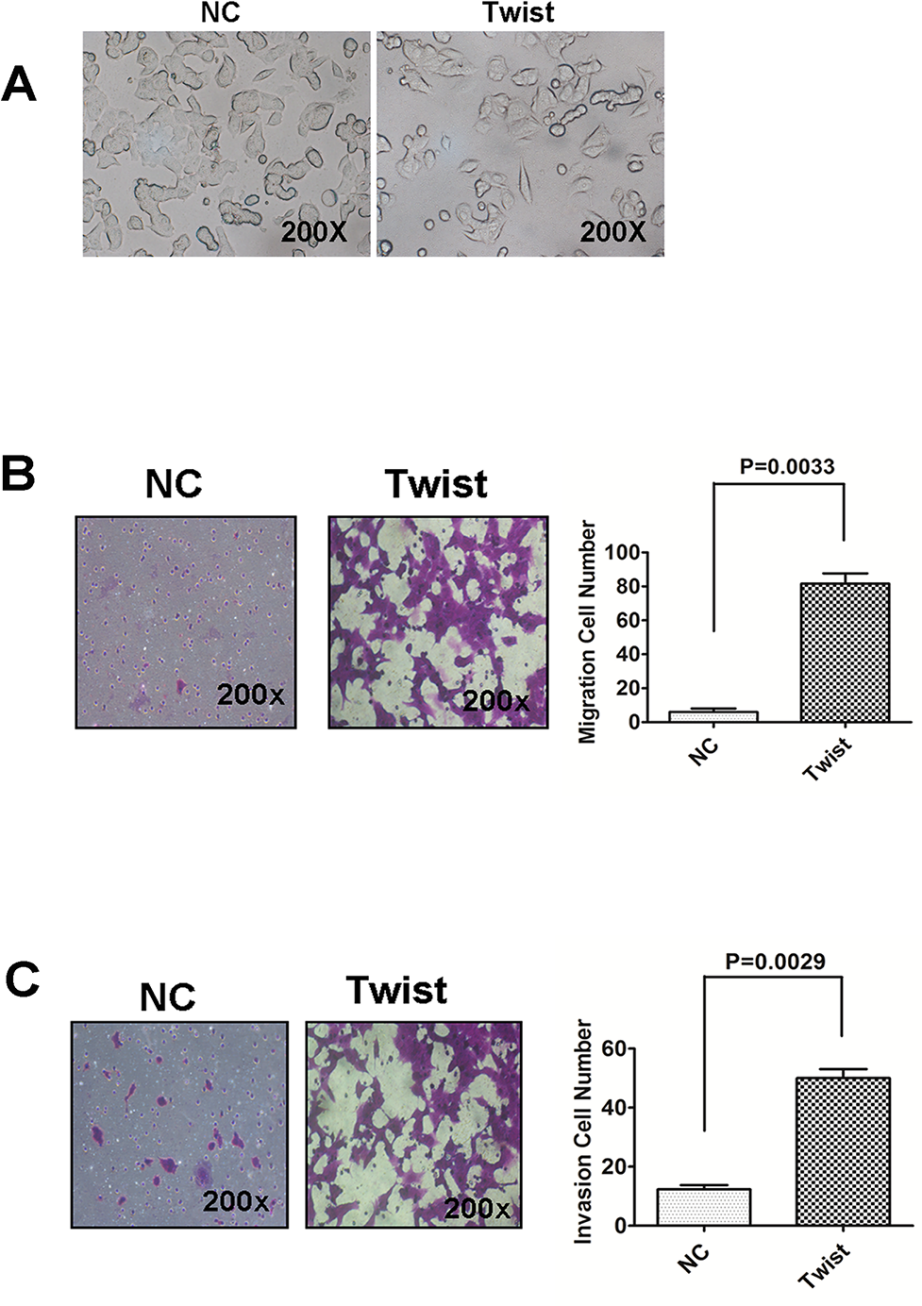
Overexpression of Twist promotes migration and invasion capability in MCF-7 cells. (A) MCF-7 cells overexpressing Twist displayed a more elongated spindle-like morphology and worse cell adhesion ability in comparison to MCF-7 cell stably transfected with pFlag-CMV as negative control. (B and C) Overexpression of Twist enhanced cell migratory and invasive capability. Empty vector and Twist-overexpressing MCF-7 cell were subjected to transwell migration and invasion assays (see Materials and Methods). Data shown are means ± SD of at least three independent experiments. *P*-values were obtained using the Student’s t-test analysis.

To investigate whether Twist overexpression promotes EMT andincreases cell proliferation, we performed morphological observation, and transwell assays (migration/invasion) in MCF-7 cells by overexpressing Twist. We observed that Twist-overexpressing MCF-7 cells displayed a more elongated spindle-like morphology and decreased cell adhesion ability (Fig 2A), one of the main characteristic features of EMT. Interestingly, overexpression of Twist enhanced migration (Fig 2B, left and middle panels) and invasion (Fig 2C, left and middle panels).The difference was statistically significant (Fig 2B and 2C, right panel).

**Fig 3:**
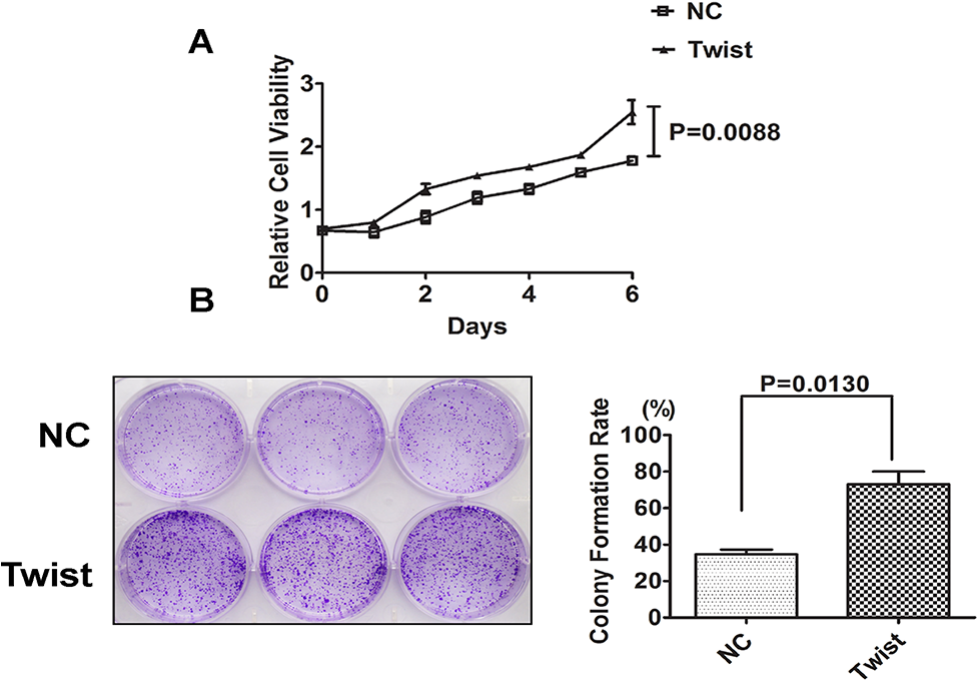
Overexpression of Twist in MCF-7 promotes proliferation and cloney formation ability. (A) Overexpression of Twist enhanced the proliferation of MCF-7 cells. CCK8 assays were used to estimate the cell proliferation at different time points. Data shown are means ± SD of at least three independent experiments. P-values were obtained using the Student’s t-test analysis. (B) Representative colony formation assay with monolayer culture to assess the tumorigenesis function of Twist. Twist-negative MCF-7 breast cancer cell were then co-transfected with pFlag-Twist and pcDNA3.1-luc2.The cell were co-transfected with pFlag-CMV and pcDNA3.1-luc2 as negative control. Twist greatly increases the colony formation of tumor cells. Quantitative analyses of colony numbers and the clony formation rate were from three independent experiments.

We further evaluate the functional significance of Twist in tumorigenesis and cell proliferationby using ectopically expressed Twist in MCF-7 and T47D cell lines. MCF-7 cells stably expressing Twist displayed enhanced proliferation ability compared to mock-transfected cells with cellular viability assay (Fig 3A). Furthermore, the colony formation ability of MCF-7-Twist cells, evaluated by monolayer culture, was significantly increased compared to mock-transfected MCF-7 cells (Fig 3B), suggesting Twist’s role in cell proliferation.

### Ectopic Twist expression promotes EMT through activation of the Akt and ERK signaling pathways

**Fig 4:**
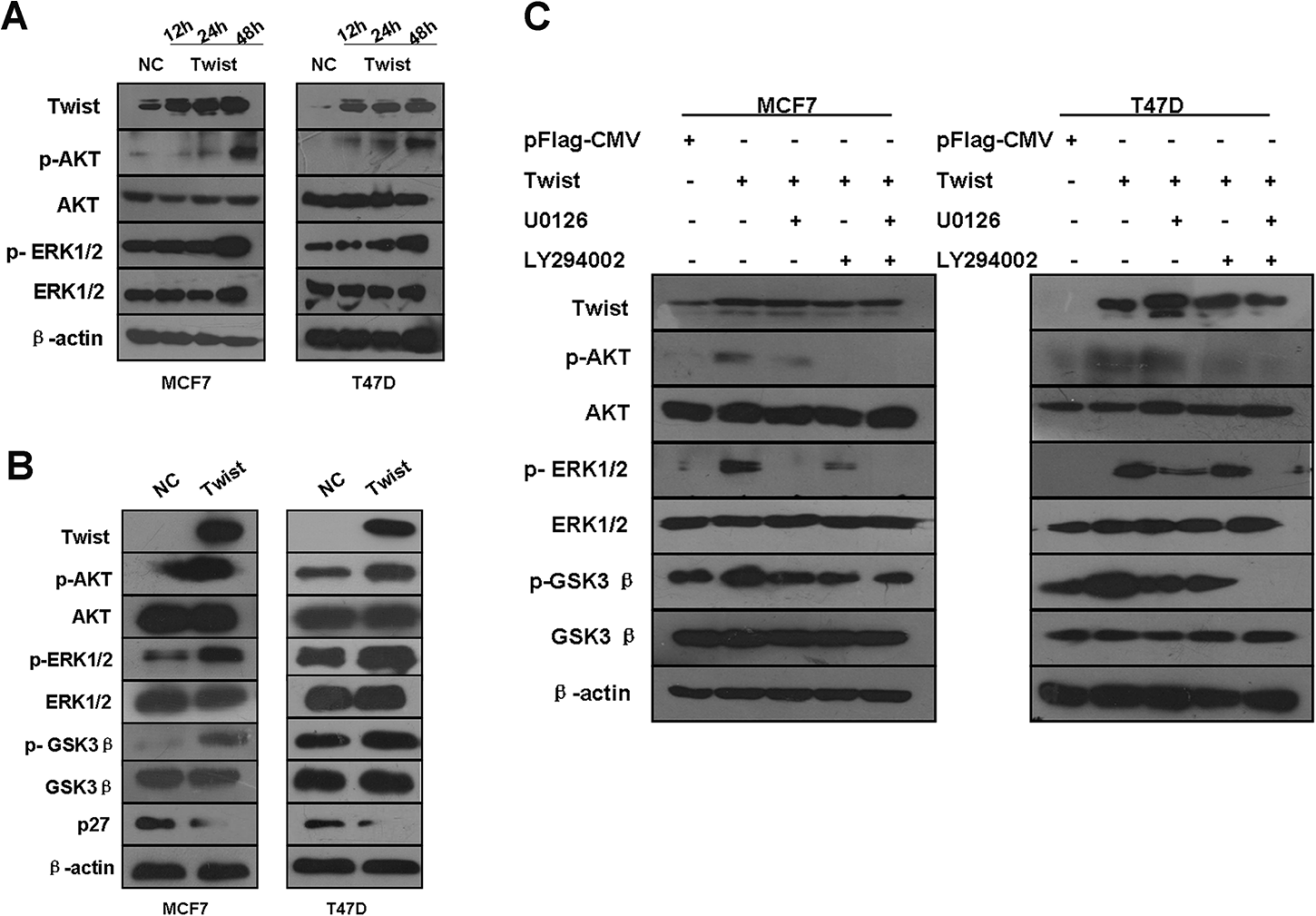
Overexpressed Twist activates Akt and ERK signaling pathways. (A) MCF-7 (left panel) and T47D (right panel) cells were transiently transfected with empty vector pFlag-CMV and pFlag-Twist, and then subjected to western blot analysis with enumerative antibodies. β-actin was used as loading control. When looked at the time course of ERK and Akt phosphorylation, the p-Akt, p-ERK expression started to increase in both MCF-7 and T47D cells at 12 or 24h after transfection with pFlag-Twist, but no change on total ERK and Akt proteins. The phosphorylation of Akt and ERK reached the highest level at 48 h in both MCF7 and T47D cell lines. (B) The down-stream proteins, total GSK3β, its phosphorylated form and p27, were detected by Western blot analysis in both MCF-7 and T47D cells at 48h after transiently expressing Twist. In parallel with increased phosphorylated Akt and ERK, phosphorylated GSK3β was increased as well as downregulation of p27. All samples were measured in triplicate. (C) ERK and Akt phosphorylation induced by over-expressed Twist were reverted by Akt or ERK inhibitor or their combination. At 48h after transfection with pFlag-Twist, MCF7-Twist and T47D-Twist cells were harvested for Western blot analysis after exposure for 6 h to either the Akt inhibitor, LY294002 (20μM), the ERK inhibitor, U0126 (20μM) or the combination of LY294002 and U0126. The administration of LY294002 or U0126 inhibited p-Akt or p-ERK, respectively. Combination administration with LY294002 and U0126 suppressed p-Akt, p-ERK simultaneously. At the same time, Serine/threonine protein kinase GSK3β, a downstream target of AKT and ERK, was also found inactivated by phosphorylation at serine 9, whereas the total GSK3β level remained unchanged.

To investigate the molecular mechanisms involved in Twist-mediated EMT, we determined whether the expression of Twist activates Akt and ERK signaling by detecting phosphorylation of Akt (Ser473) and ERK (Thr202 and Tyr204) in breast cancer cell lines. Elevated phosphorylation of Akt (Ser473) was observed in Twist-overexpressing MCF-7 and T47D cells, compared to negative control cells, while total Akt was unchanged. Moreover, Twist overexpression resulted in ERK1/2 phosphorylation at Thr-202/Tyr-204, whereas total ERK levels remained unchanged. When we looked at the time course of ERK and Akt phosphorylation, the p-Akt, p-ERK expression started to increase in both MCF-7 and T47D cells at 12 or 24h after transfected with pFlag-Twist. The phosphorylation of Akt and ERK reached the highest level at 48 h in both MCF7 and T47D cell lines (Fig 4A).

Growing evidence indicates that Snail expression is regulated by GSK3β [40, 41] a downstream target of the PI3K/AKT and ERK signaling pathways[42, 43]. GSK-3β is the major kinase regulating the protein stability and the cellular localization of Snail [41] and is inactivated by phosphorylation. Therefore, we examined whether overexpression of Twist alters the activity of GSK3β, a mediator between Twist and EMT. As shown in Fig 4A and 4B, consistent with the expected results, serine/threonine proteinkinase GSK3β, a downstream target of Twist, was also found to be inactivated by phosphorylation at serine 9 showing time dependence. This increase in the phosphorylation of GSK3β (Ser-9) indicating the inactivation of GSK3β. Similar patterns were also observed in Twist-overexpressing T47D cells. Liang *et al*. found Akt-mediated phosphorylation of p27 impairs nuclear import of p27 and blocks p27-mediated G1 arrest[44]. In order to investigate whether forced overexpression of Twist could influence the expression level of p27 resulting in promotion of cell proliferation, we determined the levels of p27 both in MCF-7-Twist and T47D-Twist cells. As expected, Western blot analysis revealed overexpression of Twist in MCF-7 and T47D cells dramatically down-regulated p27 expression (Fig 4B).

Given the earlier observations that phosphorylation of Akt (Ser473) and ERK1/2 at Thr-202/Tyr-204 were elevated in Twist-overexpressing MCF7 and T47D cells, either LY294002 or U0126 effectively inhibited phosphorylation of Akt and ERK induced by Twist overexpression (Fig 4C). Inhibitors to ERK and Akt phosphorylation (U0126 and LY294002, respectively) also inhibited the expression of their downstream targets, such as GSK3β in the MCF7-Twist and T47D-Twist cells. Furthermore, combination of LY294002 and U0126 administration dramatically suppressed p-Akt and p-ERK. Serine/threonine proteinkinase GSK3β, a downstream target of Akt or ERK, was also found to be inactivated by phosphorylation at serine 9, whereas the total GSK3β level remained changed. The Akt inhibitor (LY294002), the ERK inhibitor(U0126) or the combination of LY294002 and U0126 reversed GSK3β inactivation.

### Association of Twist expression with clinicopathological characteristics of breast cancers

**Fig 5:**
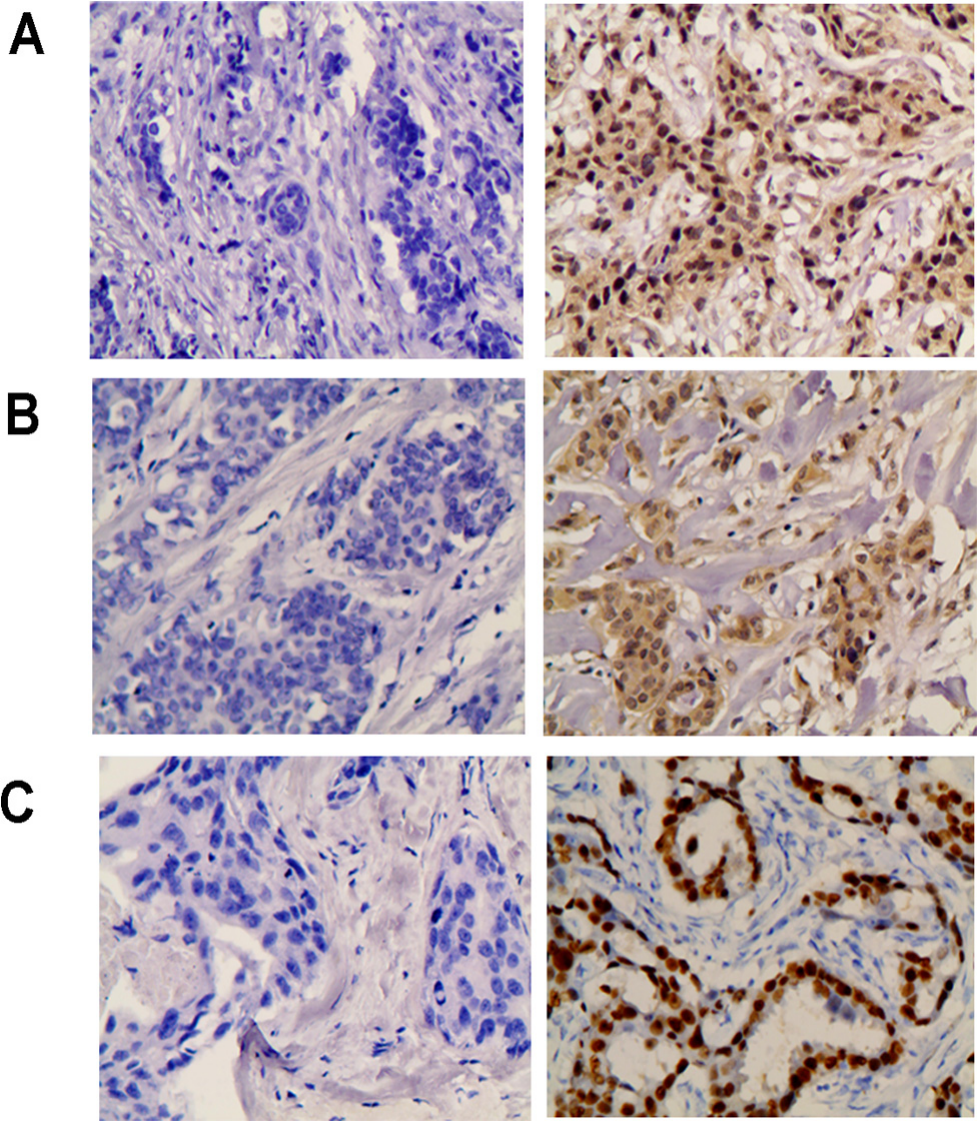
Representative images of immunohistochemical staining for Twist, p-Akt and p-ERK in invasive breast carcinomas. (A) Twist negative expression (left), and Twist positive staining (right). (B) p-Akt negative expression (left), and p-Akt positive staining (right). (C) p-ERK negative expression (left), and p-ERK positive staining (right). Original magnification, 200 ×.

To investigate the relationship between Twist expression and clinicopathological features, tissue samples from 408 patients were immunohistochemically examined for Twist expression. Of these patients, 220 (53.9%) were Twist expression-positive [Twist (+), Fig 5A], while 188 were Twist expression-negative [Twist (-)] (Table 2). Twist (+) was detected in 45.2% of stage T0~T1 patients (52/115), 54.9% of stage T2 patients (113/206) and 63.2% of stage T3~T4 patients (55/87), demonstrating that the Twist (+) frequency was significantly higher in advanced tumor stage (*P* = 0.037). Additionally, with regard to molecular subtypes, Twist expression was significantly higher in triple-negative breast cancer (55/63, 87.3%), followed by the HER2-overexpressing subtype (51/71, 71.8%), Luminal B (25/48, 52.1%) and Luminal A types (89/226, 39.4%) (*P* < 0.001). In contrast, no significant differences were seen for age, menopausal status, nodal status, TNM stage, histological type and grade.

**Table 2:** Relationship of Twist expression and clinicopathological features in breast cancer patients.

Features	Expression (%)		χ^2^	P
	Negative (n = 188)	Positive (n = 220)		
Age				
≤50	97 (46.2)	113 (53.8)	0.002	0.963
>50	91 (46.0)	107 (54.0)		
Menopausal status				
Pre	96 (44.7)	119 (55.3)	0.373	0.542
Post	92 (47.7)	101 (52.3)		
Tumor size				
T0	7 (77.8)	2 (22.2)	11.378	0.023
T1	56 (52.8)	50 (47.2)		
T2	93 (45.1)	113 (54.9)		
T3	15 (29.4)	36 (70.6)		
T4	17 (47.2)	19 (52.8)		
Nodal status				
N0	99 (50.8)	96 (49.2)	3.716	0.294
N1	42 (44.2)	53 (55.8)		
N2	28 (40.0)	42 (60.0)		
N3	19 (39.6)	29 (60.4)		
Histological grade				
I	31 (49.2)	32 (50.8)	2.508	0.285
II	79 (50.0)	79 (50.0)		
III	78 (41.9)	108 (58.1)		
Histological type				
IDC	161 (45.4)	194 (54.6)	0.580	0.446
Non-IDC	27 (50.9)	26 (49.1)		
ER				
Negative	34 (23.1)	113 (76.9)	48.709	<0.001
Positive	154 (59.0)	107 (41.0)		
PR				
Negative	53 (29.4)	127 (70.6)	35.870	<0.001
Positive	135 (59.2)	93 (40.8)		
HER2				
Negative	145 (50.2)	144 (49.8)	6.686	0.010
Positive	43 (36.1)	76 (63.9)		
Molecular subtypes				
Luminal A	137(60.6)	89(39. 4)	56.716	<0.001
Luminal B	23 (47.9)	25 (52.1)		
HER2-enriched	20 (28.2)	51 (71.8)		
TNBC	8 (12.7)	55 (87.3)		

* IDC: invasive ductal carcinoma.

The relationship between Twist and other biomarkers is shown in Table 2 and Table 3. Out of 408 patients, 64.0% (261/408) were ER-positive, 55.9% (228/408) were PR-positive, and 29.2% (119/408) were HER2-positive. Compared to ER-negative group, patients in the ER-positive group were more likely lack Twist expression [Twist (+) frequency: 41.0% in ER-positive tumors vs. 76.9% in ER-negative tumors, *P* < 0.001]. Similar results were found for PR-positive tumors [Twist (+) frequency: 40.8% in PR-positive tumors vs. 70.6% in PR-negative tumors, *P* < 0.001]. In contrast, patients in the HER2-positive group were inclined to be positive for Twist expression [Twist (+) frequency: 63.9% in HER2-positive tumors vs. 49.8% in HER2-negative tumors, *P* = 0.010], which is consistent with the relationship between Twist and the molecular subtypes mentioned above. For other markers, 70.6% (288/408) were positive for VEGF-C, 58.1% (237/408) were positive for p53, 86.0% (351/408) displayed elevated Ki67, and 80.4% (328/408) displayed E-cadherin. Twist (+) rate was significantly higher in VEGF-C-positive group, p53-positive group and Ki67-positive group than that of VEGF-C-negative group (59.7% vs. 40.0%, *P* < 0.001), p53-negative group (62.4% vs. 42.1%, *P* < 0.001) and Ki67-negative group (59.3% vs. 21.1%, *P* < 0.001). Nevertheless, Twist (+) was less frequent in the E-cadherin-positive group than in the E-cadherin-negative group (45.4% vs. 88.8%, *P*<0.001).

**Table 3:** Association of Twist expression with other molecules in breast cancer patients.

Biomarker	Twist expression (%)		χ^2^	P
	Negative (n = 188)	Positive (n = 220)		
VEGF-C				
Negative	72 (60.0)	48 (40.0)	13.261	<0.001
Positive	116 (40.3)	172 (59.7)		
p53				
Negative	99 (57.9)	72 (42.1)	16.543	<0.001
Positive	89 (37.6)	148 (62.4)		
Ki67				
Negative	45 (78.9)	12 (21.1)	28.810	<0.001
Positive	143 (40.7)	208 (59.3)		
E-cadherin				
Negative	9 (11.2)	71 (88.8)	48.583	<0.001
Positive	179 (54.6)	149 (45.4)		

Twist, p-ERK and p-Akt were determined by immunohistochemistry in 102 cases of breast cancers (Fig 5A–5C). We demonstrated a positive correlation of Twist expression and p-Akt (R = 0.207, *P* = 0.039) or p-ERK *(*R = 0.200, *P* = 0.046) expression, using the Spearman's Rank Correlation Test.

### Effect of Twist expression and clinicopathological features on DFS and OS

**Fig 6:**
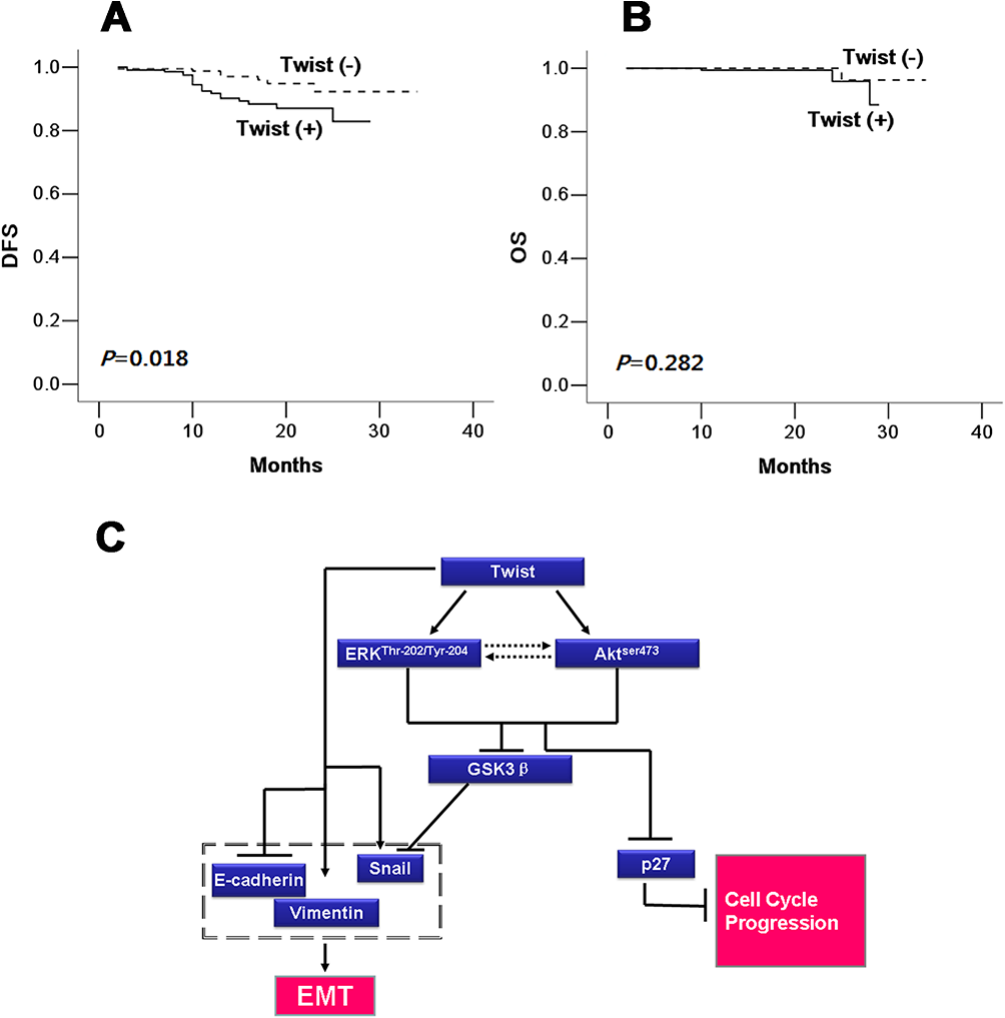
Twist expression predicts worse DFS and OS in patients with breast cancer by Kaplan-Meier analysis, and a schematic diagram of the proposed model. (A) The left side indicates DFS in the Twist (+) group was lower than that of Twist (-) group (*P* = 0.018). (B) The right side demonstrates there was no difference in OS between Twist (+) and Twist (-) groups (*P* = 0.282). Twist (+): Twist-positive group; Twist (-): Twist-negative group. (C) A schematic diagram of the proposed model. A proposed model to illustrate how Twist activates both Akt and ERK pathways through their phosphorylation, further down-regulates phsopho-GSK-3β and p27, and in turn induces the expression of EMT marker properties associated with EMT.

To investigate the relevance of clinicopathological features and biomarkers reported here, we analyzed DFS and OS for breast cancer patients, using the Kaplan-Meier log-rank test. DFS and OS were calculated for 2 to 34 months, with a median period of 15 and 16 months. As shown in Table 3, for DFS, the Twist (+) group tended to have a poorer prognosis than the Twist (-) group (30-month DFS rate: 82.9% vs. 92.3% *P* = 0.018, Fig 6A). For clinicopathological features, the 30-month DFS frequency decreased along with tumor size (100.0% in the T0 group, 99.1%, 87.3%, 76.4% and 74.2% in the T1, T2, T3 and T4 groups, respectively; *P* = 0.013). Nodal status (94.4%, 92.0%, 73.3% and 73.3% in the N0, N1, N2 and N3 groups, respectively; *P* < 0.001) and TNM stage (100.0%, 98.8%, 94.2% and 74.7% in TNM0, TNM1, TNM2, and TNM3 stages, respectively; *P* < 0.001). Moreover, patients with well-differentiated tumors held a higher 30-month DFS rate (100%) than those with moderately or poorly differentiated tumors (90.9% and 80.0%, *P* = 0.003). For other biomarkers, the 30-month PFS rate was significantly lower in the VEGF-C/p53-positive group than that in the VEGF-C (84.7% vs. 95.9%, *P* = 0.042)/p53-negative group (84.7% vs. 90.9%, *P* = 0.036, Table 4). Only positive E-cadherin expression indicated better DFS compared to negative E-cadherin expression (30-month DFS rate: 90.9% vs. 77.5%, *P* = 0.023). Adversely, age, menopausal status, morphology, molecular subtypes (ER-, PR-, HER2- and Ki67-positive) were not predictors for DFS. Otherwise, the 30-month OS of the Twist-positive group tended to be lower than that of Twist negative group (88.5% vs. 96.3%), but failed to meet statistical significance (*P* = 0.282, Fig 6B, Table 4). Among all these clinicopathological features and biomarkers, only ER (*P* = 0.047) and E-cadherin (*P* = 0.042) were negative predictors for OS.

**Table 4:** Kaplan-Meier analysis for prediction of PFS and OS.

Characteristic	PFS (%)			OS (%)		
	15 Months	30 Months	P	15 Months	30 Months	P
Age						
≤50	92.2	92.2	0.706	99.4	96.3	0.960
>50	93.6	82.4		100.0	89.1	
Menopausal status						
Pre	91.6	87.3	0.735	99.4	90.0	0.384
Post	94.3	87.1		100.0	95.8	
Tumor size						
T0	100.0	100.0	0.013	100.0	100.0	0.219
T1	99.1	99.1		100.0	100.0	
T2	92.2	87.3		99.4	99.4	
T3	82.3	76.4		100.0	78.8	
T4	91.4	74.2		100.0	75.0	
Nodal status						
N0	97.9	94.4	<0.001	100.0	100.0	0.090
N1	94.8	92.0		100.0	100.0	
N2	90.5	73.3		100.0	68.8	
N3	76.2	73.3		97.7	89.5	
Histological grade						
I	100.0	100.0	0.003	100.0	100.0	0.060
II	94.3	90.9		100.0	100.0	
III	89.3	80.0		99.3	81.4	
Histological type						
IDC	92.6	89.0	0.790	99.6	95.6	0.592
Non-IDC	94.9	78.4		100.0	83.3	
ER						
Negative	91.4	83.0	0.279	99.1	80.2	0.047
Positive	93.8	89.4		100.0	97.2	
PR						
Negative	91.3	85.5	0.363	99.2	88.3	0.226
Positive	94.2	88.8		100.0	96.4	
HER2						
Negative	94.7	89.6	0.121	99.5	94.5	0.867
Positive	88.4	81.0		100.0	85.7	
Molecular subtypes						
Luminal A	95.2	90.0	0.325	100.0	96.7	0.118
Luminal B	95.3	89.1		100.0	100.0	
HER2-enriched	92.6	88.4		100.0	75.0	
TNBC	88.3	75.7		98.0	85.7	
Twist						
negative	97.0	92.3	0.018	100.0	96.3	0.282
positive	89.3	82.9		99.4	88.5	
VEGF-C						
Negative	98.1	95.9	0.042	100.0	100.0	0.347
Positive	90.8	84.7		99.5	91.5	
p53						
Negative	96.7	90.9	0.036	100.0	92.4	0.635
Positive	90.0	84.7		99.4	95.1	
Ki67						
Negative	98.2	91.8	0.487	100.0	91.7	0.914
Positive	92.0	86.3		99.6	92.8	
E-cadherin						
Negative	88.3	77.5	0.023	98.6	84.0	0.042
Positive	94.2	90.9		100.0	97.2	

* IDC: invasive ductal carcinoma.

## Discussion

Twist, a transcriptional factor, promotes epithelial-mesenchymal transition (EMT) in variety of malignancies, such as breast[16], lung[17], prostate cancers[18, 19], and gastric carcinoma[20]. In particular, Yang *et al*. has shown that Twist overexpression in breast cancer can induce and promote tumorigenesis[21]. Since then, many studies have focused on the role of Twist on the progression and metastasis of malignancies. For instance, Martin[16] and Watanabe[30] have demonstrated the higher expression of this protein in human breast cancer tissues. However, the mechanism regarding how Twist mediates breast cancer development and progression remains largely unknown. In this study, we immunohistochemically determined Twist expression in 408 patients with breast cancers, and found a 54% positive frequency, similar to that reported previously. Moreover, we show that Twist is highly expressed in tumors with large size, and positively associates with expression of HER2, an epidermal growth factor receptor. This part of the results are similar to Huang et al’s findings that when compared to low expression, high serum and tissue levels of MMP-2 and Twist were associated with lymph node metastasis and higher TNM stage[45]. Further more we demonstrated a positive correlation of Twist expression and p-Akt and p-ERK expression, when a percent of positively stained cells was used in human breast cancer tissues. Conversely, Twist is negatively associated with ER and/or PR expression. The inverse correlation between Twist and hormone receptors in human cancers is also supported by *in cellulo* findings that Twist could transcriptionally down-regulate ER expression via binding to the ERα promoter [46]. van Nes JG et al found that co-expression of Snail-Twist was associated with low-E-cadherin and high-N-cadherin expression, especially in ER-positive tumors, suggesting that, through interactions with ER, Snail and Twist may regulate E- and N-cadherin expression, thereby inducing EMT[47]. These findings together strongly support Twist’s role in inhibiting the luminal phenotype.

In addition, p53 and Ki67 were also analyzed to look for a relationship with Twist. In this cohort, p53 positivity was associated with cytoplasmic expression of Twist, keeping consistent with previous reports [48]. Moreover, Ki67 immunostaining was also associated with Twist overexpression, suggesting Twist’s role as an oncogene to promote proliferation.

In terms of the mechanism underlying Twist regulates EMT and proliferative capability regulation by Twist, a few oncogenic molecular pathway have been extensively studied. For instance, PI3K/AKT signaling pathways have been demonstrated to be a downstream of Twist in several types of malignancies [49–51]. Herein, we showed that Twist, when overexpressed, can trigger phosphorylation of Akt, supporting the role of Twist-mediated activation of PI3K/AKT pathway. Twist has been shown to activate the Akt signaling pathway by inducing the expression of Akt [44]. In the present study, however, we detected the elevated phosphorylation of Akt (active part of total Akt) in cells, but not of total Akt, suggesting its post-translational regulation of Akt. Furthermore, we showed that exogenous expression of Twist activates the MEK/ERK signaling pathway, in addition to activation of Akt. This regulation induces cellular responses leading to the conversion of epithelial cells into invasive mesenchymal cells. Thus, Twist indeed promotes growth and plays an important role in tumorigenesis, as previously reported.

As a trigger for EMT, we further illustrate that enhanced Twist expression increases cell migration and invasion. Morphologically, cells overexpressing Twist show a conversion to cells with mesenchymal properties, including expression of vimentin, and decreases in E-cadherin. Conversely, in previous reports, knockdown of this protein significantly suppressed the progression of breast cancer metastases in MDA-MB-231 cells, suggesting sufficiency for the maintenance of a mesenchymal phenotype in breast cancer cells. It might be necessary to see the effects of inhibiting Twist on migration/invasion, and tumorigenesis in further study.

Interestingly, when further looked into ERK and Akt’s downstream genes, p-GSK3β was up-regulated and p27 protein was down-regulated by overexpression of Twist. Those effects were reverted by suppressing either ERK or Akt or both by small molecule inhibitors, suggesting Twist’s EMT through, at least in part, activation of ERK and/or Akt pathway. Therefore, we proposed a mechanistic model to illustrate how Twist activates both Akt and ERK pathways through their phosphorylation, further up-regulates phsopho-GSK-3β and down-regulates p27, and in turn induces EMT (Fig 6C).

In conclusion, our results demonstrate Twist expression can increase proliferative, transforming, migratory and invasive capability, strongly suggesting that Twist plays an important role in tumorigenesis and progression of breast cancer and promotes EMT, at least in part, through activation of the Akt and ERK signaling pathways. In particular, Twist expression was shown to associate with worse prognosis for both DFS and OS, indicating its role as an independent prognostic factor in Chinese breast cancer patients. Thus, taken together, our data demonstrate that Twist promotes tumor progression of breast cancers and may serve as a potential therapeutic target in the future.
